# Insights into the role of legionella effectors on host metabolic perturbations

**DOI:** 10.3389/fcimb.2024.1458276

**Published:** 2024-09-11

**Authors:** Zhihao Wang, Lei Song, Jingai Che, Chunxiuli Li

**Affiliations:** ^1^ Department of Geriatrics, The First Hospital of Jilin University, Changchun, China; ^2^ Department of Respiratory Medicine, Center for Pathogen Biology and Infectious Diseases, The First Hospital of Jilin University, Changchun, China; ^3^ Department of Respiratory Medicine, Meihekou Central Hospital, Meihekou, China

**Keywords:** type IV secretion system, glucose, phosphoinositide, virulence, protein synthesis

## Abstract

*Legionella* infection, the causative agent of Legionnaires’ disease, represents a significant threat to human health. The pathogenesis of this infection is intricately linked to the complex interactions between the bacterium and its host, resulting in profound metabolic perturbations. Central to these metabolic shifts is the bacterium’s modulation of lipid metabolism, with changes in lipid synthesis and breakdown modifying membrane composition and function. These alterations can influence cellular signaling and immune responses, further contributing to disease progression. It also disrupts glucose utilization and lipid metabolism, altering cellular energy production and immune responses. Additionally, *Legionella* infection perturbs amino acid and protein metabolism, affecting protein synthesis and degradation, leading to changes in cellular functions and immune responses. This mini-review underscores the complexity of metabolic perturbations in *Legionella* infection and their significance in host-pathogen interactions. Understanding these metabolic shifts provides valuable insights into the pathogenesis of Legionnaires’ disease and could lead to the development of novel therapeutic strategies.

## Introduction

*Legionella*, a bacterium that commonly colonizes aquatic environments, has emerged as a significant public health concern due to its ability to cause Legionnaires’ disease, a severe form of pneumonia (Graham et al., 2022). The pathogenicity of *Legionella* is multifaceted, involving complex interactions with the host immune system and metabolic systems (Luo et al., 2021; Yang et al., 2023). Among these, the metabolic perturbations triggered by *Legionella* infection are particularly noteworthy as they play a pivotal role in disease progression and pathogenesis.

The host’s metabolic homeostasis is a highly regulated process, ensuring the efficient conversion of nutrients into energy and the synthesis of macromolecules required for cellular growth and function. However, *Legionella* infection disrupts this homeostasis, leading to significant metabolic perturbations that favor bacterial survival and replication while compromising host cellular functions. These metabolic shifts are not limited to a single biochemical pathway but involve multiple interconnected systems. For instance, *Legionella* infection is known to perturb carbohydrate metabolism, altering glucose utilization, which in turn affects cellular energy production (Eisenreich and Heuner, 2016). Similarly, amino acid and protein metabolism are also disrupted, leading to changes in protein synthesis and degradation, further modifying cellular functions and immune defenses (Schunder et al., 2014; Belyi, 2020; Belyi et al., 2022). Furthermore, lipid metabolism is significantly affected by *Legionella* infection. Changes in lipid synthesis and breakdown can modify membrane composition and function, thereby influencing cellular signaling and the replicative niche of the bugs (Swart and Hilbi, 2020; Kowalczyk et al., 2021). These alterations contribute to the pathogenesis of Legionnaires’ disease and offer valuable insights into the complex host-pathogen interactions.

In this mini-review, we delve into the metabolic alterations triggered by *Legionella* infection, focusing on the specific biochemical pathways and mechanisms involved. We aim to provide a comprehensive understanding of how these metabolic perturbations contribute to disease progression and pathogenesis, and how they can be exploited for the development of novel therapeutic strategies. By doing so, we hope to gain deeper insights into the host-pathogen interactions that underlie *Legionella* infection and its associated diseases.

## The type iv secretion system of *Legionella*

A distinctive feature of *Legionella* is its possession of the T4SS, a complex molecular machinery that enables intimate interactions with host cells (Durie et al., 2020; Bock et al., 2021). The T4SS serves as a crucial tool for *Legionella* to deliver effector proteins into the interior of host cells. These effector proteins play vital roles in the bacterium’s pathogenic process, manipulating various physiological activities of the host cell to aid in evasion of the immune system and successful reproduction (Song et al., 2021; Song et al., 2022; Fu et al., 2022b). Through this mechanism, *Legionella* establishes a favorable environment for its survival and multiplication within the host cell (Iyer and Das, 2021).

Specifically, the T4SS consists of multiple subunits that work synergistically to form a channel spanning the bacterial inner and outer membranes (Iyer and Das, 2021). This channel allows *Legionella* to directly transport its synthesized effector proteins into the interior of host cells. This direct delivery method enables the bacterium to quickly and effectively influence host cell functions, thereby achieving its pathogenic goals. The T4SS exhibits high specificity and selectivity, enabling precise targeting of effector proteins. *Legionella* can precisely select the effector proteins to be delivered and ensure their accurate targeting to specific locations within the host cell (Urbanus et al., 2016; Fu et al., 2022a, Fu et al., 2022b). This specificity ensures effective manipulation of the host cell without causing excessive damage. In conclusion, the T4SS of *Legionella* represents a complex and intricate molecular machinery that enables intimate interactions and manipulation of host cell functions. This mechanism not only enhances the bacterium’s pathogenic capabilities but also provides new insights into the understanding of the interaction between bacteria and host cells.

## Role of *Legionella* Icm/Dot substrates in phosphoinositide metabolism

*Legionella* effectors represent a sophisticated arsenal of virulence factors that target host phospholipid metabolism at multiple levels. By degrading, binding, or modifying specific phospholipids, these effectors disrupt membrane integrity, trafficking, and signaling, ultimately favoring *Legionella*’s infection and persistence within host cells. The integrated impact of these effectors is likely to be synergistic, as they target different aspects of phospholipid metabolism and membrane function. One such effector protein, MavQ, has recently been identified as a key player in the regulation of phosphatidylinositol (PtdIns) metabolism within the LCV (Hsieh et al., 2021; Li et al., 2021). MavQ, exhibits a unique enzymatic activity: it specifically phosphorylates PtdIns to generate phosphatidylinositol 3-phosphate (PtdIns3P) (Hsieh et al., 2021; Li et al., 2021). This activity is significant because PtdIns3P is an essential intermediate in the PtdIns phosphorylation cascade, leading to the production of more complex phosphoinositides like PtdIns4P (Li et al., 2021). Once PtdIns3P is generated by MavQ, it serves as a substrate for the subsequent actions of LepB (Dong et al., 2016). LepB, another Dot/Icm effector, exhibits phosphatidylinositol 4-kinase activity, catalyzing the phosphorylation of PtdIns3P to PtdIns3,4P (Dong et al., 2016). By acting downstream of LepB, the effector protein SidF functions as a phosphatidylinositol 3-phosphate phosphatase, converting PtdIns3,4P to PtdIns4P, which completes the biosynthetic pathway of PtdIns4P on the LCV membrane (Hsu et al., 2012; Dong et al., 2016).

Some other effector including SidP, LppA, VipD, PlcC, and LpdA, are Icm/Dot substrates delivered by *Legionella* pneumophila into host cells, where they play crucial roles in modulating phosphoinositide metabolism. SidP, a phosphatidylinositol 3-phosphatase, can convert PtdIns3,5P to PtdIns5P and PtdIns3P to PtdIns *in vitro*, although its activity in infected cells remains unclear (Toulabi et al., 2013). LppA, a CX5R motif PI phosphatase, hydrolyzes specific phosphoinositide species to produce PtdIns4P *in vitro*, but does not seem to significantly alter the PI pattern of *Legionella*-containing vacuoles (LCVs) in live cells (Weber et al., 2014). VipD, on the other hand, is a lipase that hydrolyzes PtdIns3P, and intriguingly, binds to Rab5 and Rab22 (Gaspar and Machner, 2014). This effector removes PtdIns(3)P from endosomal membranes, potentially promoting the evasion of the endocytic pathway by LCVs (Gaspar and Machner, 2014). PlcC, a metallophospholipase C, exhibits broad lipid hydrolyzing activity, including phosphatidylcholine (PC), phosphatidylglycerol (PG), and PtdIns (Aurass et al., 2013). Lastly, LpdA, a phospholipase D, hydrolyzes PG, PtdIns, PtdIns3P, and PtdIns4P to yield phosphatidic acid (PA) (Schroeder et al., 2015). Through these enzymatic activities, LpdA likely alters membrane properties and signaling cascades within LCVs (Schroeder et al., 2015). By modulating the PI composition and signaling cascades within LCVs, these effectors likely play important roles in *Legionella*’s pathogenesis, including the evasion of host defenses, modulation of membrane trafficking, and the establishment of a replicative niche within host cells. Further research is needed to fully elucidate the precise mechanisms and functional outcomes of these effectors within the context of *Legionella* infection.

**Figure 1:**
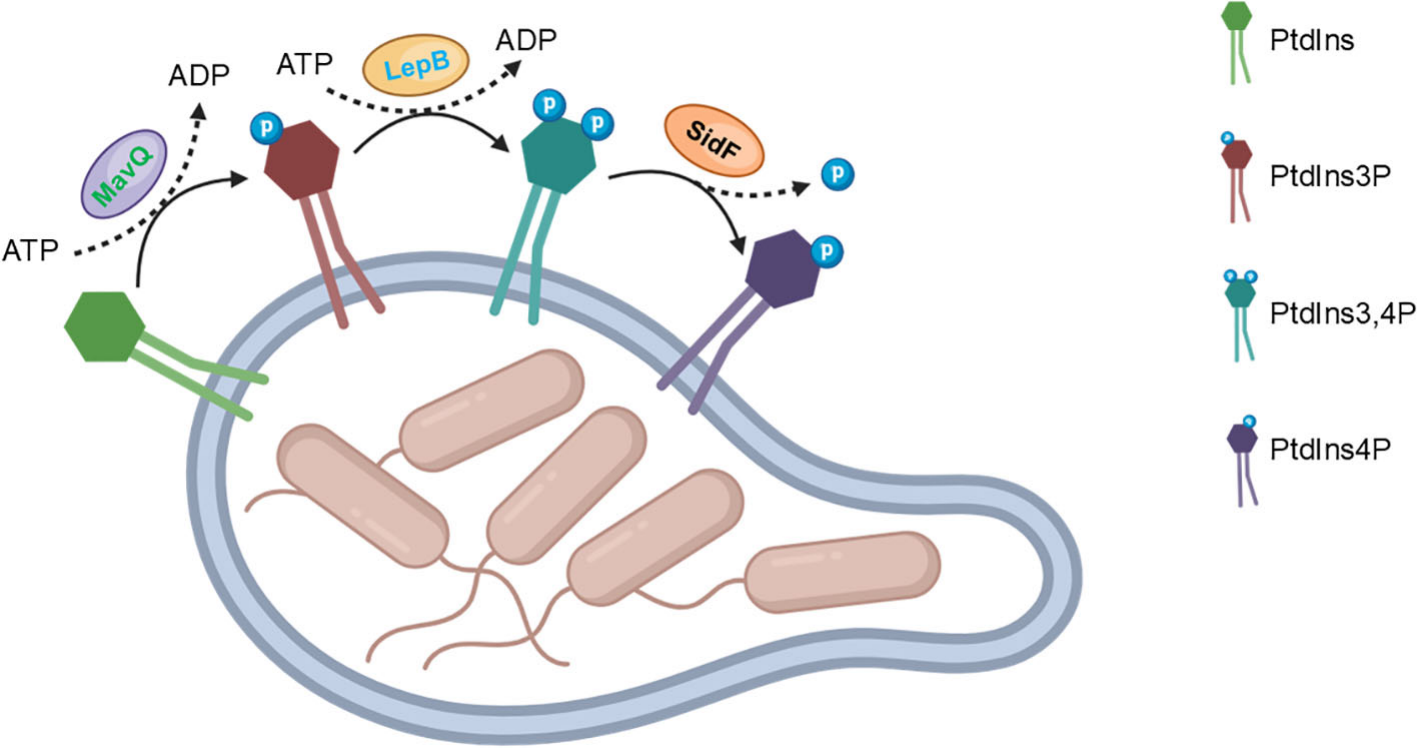
*Legionella* effectors synergistically convert PtdIns to PtdIns4P.

In summary, the large cohort effectors play crucial roles in the regulation of PtdIns metabolism within the LCV. By targeting specific steps in the PtdIns phosphorylation cascade, these effectors generate PtdIns4P, which serves as an anchor for other Dot/Icm effectors (**Figure 1**). This process is essential for *Legionella*’s ability to establish and maintain infection within host cells. Further research into the mechanisms and functions of these effectors may provide new insights into *Legionella*’s pathogenesis and potential therapeutic targets for the treatment of Legionnaires’ disease.

## The impact of *Legionella* effectors on host protein synthesis

The impact of *Legionella* effectors on host cell protein synthesis is multifaceted and profoundly disrupts the normal cellular processes. Several effector proteins produced by *Legionella*, including Lgt1, Lgt2, Lgt3, SidI, LegK4, SidL, and RavX, have been identified to play a crucial role in inhibiting protein synthesis in infected host cells (Belyi et al., 2022). Among these, Lgt1-3 and SidI target elongation factors, the key components of the protein synthesis machinery (Shen et al., 2009; Sol et al., 2019). Lgt1-3 are members of the GT-A type glucosyltransferase family that mono-glucosylate elongation factor eEF1A (Belyi et al., 2006; Tzivelekidis et al., 2011). This glucosylation of eEF1A leads to its inactivation, thereby inhibiting protein synthesis (Belyi et al., 2006). Interestingly, SidI binds to both eEF1A and its guanine nucleotide exchange factor eEF1Bg, further disrupting their interaction and resulting in the inhibition of protein synthesis (Shen et al., 2009).

LegK4, another *Legionella* effector, functions as a protein kinase that phosphorylates Hsp70 proteins. Hsp70 proteins are involved in various cellular processes, including protein folding, translocation, and degradation (Moss et al., 2019). By phosphorylating Hsp70 proteins, LegK4 disrupts their function and ultimately inhibits protein synthesis (Moss et al., 2019). While SidL and RavX have not yet been assigned specific targets in the protein synthesis machinery, they are likely to affect protein synthesis indirectly by modulating other cellular processes (Subramanian et al., 2023). This suggests that *Legionella* effectors may have pleiotropic effects, targeting multiple cellular processes to promote infection.

The inhibition of protein synthesis by *Legionella* effectors has profound consequences for the host cell. It disrupts the normal functioning of the cell, impairs its ability to respond to stress, and ultimately leads to cell death. This allows *Legionella* to evade host immune defenses, replicate within the cell, and spread to other parts of the body. Further research into the mechanisms and functions of these effectors could provide novel insights into *Legionella* pathogenesis and potential therapeutic targets.

## Conclusion and prospective

Metabolic perturbations in *Legionella* infection provide deep insights into the complex host-pathogen interactions that underlie the pathogenesis of Legionnaires’ disease. The identification and characterization of *Legionella* effectors such as SidP, LppA, VipD, PlcC, and LpdA that target specific phosphoinositide species within host cells reveal a sophisticated mechanism of how the pathogen modulates membrane trafficking, alters signaling pathways, and establishes a favorable replicative niche. These effectors exhibit diverse enzymatic activities that perturb the host cell’s metabolic landscape, including the hydrolysis of phospholipids and PtdIns, altering membrane properties, and disrupting cellular signaling cascades. These perturbations enable *Legionella* to evade host immune defenses, hijack host cellular processes, and ultimately cause disease.

Future research in this area is poised to yield exciting new insights into *Legionella* pathogenesis and host-pathogen interactions. Understanding these interactions could provide novel targets for therapeutic intervention. The development of new diagnostic tools and therapeutic strategies based on the understanding of Legionella pathogenesis could significantly improve the management of Legionnaires’ disease. By investigating the synergistic actions of bacterial effectors, the broader impact of Legionella infection on host cell metabolism, and the interactions with host genetics, we are optimistic that continued research will lead to more effective treatments and better patient outcomes, reducing the morbidity and mortality associated with this infection.
