# Supplementary material for: Insights into the role of legionella effectors on host metabolic perturbations
Source: Front Cell Infect Microbiol. 2024 Sep 11;14:1458276. doi: 10.3389/fcimb.2024.1458276 (PMC11422348; doi:10.3389/fcimb.2024.1458276)
Supplement: Supplementary file 1 [file Table1.docx]

**TABLE 1 *L. pneumophila* Dot/Icm effectors involved in host metabolic pathway.**

| **Effectors (Gene ID)** | **Aliases** | **Interactor/Substrate** | **Enzymatic activity** | **Function** | **References** |
| --- | --- | --- | --- | --- | --- |
| **Metabolic pathway** | | | | | |
| **Lpg2975** | MavQ | PtdIns | phosphatidylinositol 3-kinase | phosphorylation of PtdIns | Hsieh et al., 2021;Li et al., 2021 |
| **Lpg2490** | LepB | PtdIns3P | phosphatidylinositol 4-kinase | phosphorylation of PtdIns3P | Dong et al., 2016 |
| **Lpg2584** | SidF | PtdIns3,4P | phosphatidylinositol 3-phosphate phosphatase | converting PtdIns3,4P to PtdIns4P | Hsu et al., 2012; Dong et al., 2016 |
| **Lpg0130** | SidP | PtdIns3,5P and PtdIns3P | phosphatidylinositol 3-phosphate phosphatase | converting PtdIns3,5P to PtdIns5P and PtdIns3P to PtdIns | Toulabi et al., 2013 |
| **Lpg2819** | LppA | PtdIns3,4P, PtdIns4,5P and PtdIns3,4,5P | phytase | converting Phosphoinositides to PtdIns4P | Weber et al., 2014 |
| **Lpg2831** | VipD | PtdIns3P | phospholipase A1 | removal of PtdIns3P from endosomal membranes | Gaspar and Machner, 2014 |
| **Lpg0012** | PlcC | PC, PG, and phosphatidylinositol | phospholipase C | Hydrolyzing PC, PG, and phosphatidylinositol | Aurass et al., 2013 |
| **Lpg1888** | LpdA | PG, PtdIns, PtdIns3P, and PtdIns4P | Phospholipase D | hydrolyzing PG, PtdIns, PtdIns3P, and PtdIns4P to yield PA | Schroeder et al., 2015 |
| **Protein synthesis machinery** | | | | | |
| **Lpg1368** | Lgt1 | eEF1A | glucosyltransferase | Inhibition of protein synthesis | Shen et al., 2009;Sol et al., 2019 |
| **Lpg2862** | Lgt2 | eEF1A | glucosyltransferase | Inhibition of protein synthesis | Shen et al., 2009;Sol et al., 2019 |
| **Lpg1488** | Lgt3 | eEF1A | glucosyltransferase | Inhibition of protein synthesis | Shen et al., 2009;Sol et al., 2019 |
| **Lpg2504** | SidI | eEF1A and eEF1Bg | Unknown | Inhibition of protein synthesis | Shen et al., 2009;Sol et al., 2019 |
| **Lpg0208** | LegK4/Pkn5 | Hsp70 | Ser/Thr kinase | Inhibition of host translation | Moss et al., 2019 |
| **Lpg0437** | Ceg14/SidL | unknown | Unknown | Affecting protein synthesis | Fontana et al., 2011 |
| **Lpg1489** | RavX | unknown | Unknown | Affecting protein synthesis | Barry et al., 2013 |
